# Transforming Clinical Skills Training: Integrating OSCE into Team-Based Learning for teaching undergraduate medical students

**DOI:** 10.12669/pjms.42.6.12667

**Published:** 2026-06

**Authors:** Ayesha Akram, Ahsan Sethi

**Affiliations:** 1 Ayesha Akram, Associate Professor, Gynaecology & Obstetrics, HITEC-Institute of Medical Sciences (IMS), Taxila, Pakistan; 2 Ahsan Sethi, Associate Professor, Health Professions Education, Department of Public Health, College of Health Sciences, QU Health, Qatar University, Doha, Qatar

**Keywords:** Clinical skills, OSCE, Team-based Learning, Undergraduate Medical Students

## Abstract

**Objective:**

Team-Based Learning (TBL) approach primarily targets the cognitive domain and employs MCQs during iRAT and tRAT stages to promote active learning and problem-solving through collaborative teamwork. We adapted TBL for teaching the psychomotor domain by replacing MCQs with OSCEs during iRAT and tRAT and introducing self-assessment followed by feedback from peers and tutors on clinical skills performance. We assessed the effectiveness of adapted TBL approach on clinical skills performance through comparison with standard clinical teaching.

**Methodology:**

A Quasi-experimental study with a crossover design was carried out from August 2021-May 2022 at HITEC-Institute of Medical Sciences, Pakistan. The study involved two skills stations: obstetric examination & eclampsia drill. The 4^th^ Year MBBS students (n=80) were randomly assigned to control and experimental groups. The control group underwent standard clinical skills training, while the experimental group underwent an adapted TBL learning experience with OSCEs during iRAT and tRAT. The resource materials were provided for both groups and both groups had the opportunity for feedback. Participants’ performance was assessed using pre and post-test OSCEs. Descriptive statistics were reported as mean±SD and inferential statistics were applied to evaluate the statistical significance of changes in scores.

**Results:**

The mean post-test scores of the experimental groups in both OSCE stations were significantly higher than those of the control groups (13.96±0.62 vs 10.34±0.61, p<0.001) and (13.60±0.52 vs 8.45±0.42, p<0. 001. There was no significant difference in the mean pre-test scores.

**Conclusions:**

Adapted TBL integrated with OSCE enhances clinical skill development in undergraduate medical students. The approach encourages both individual and team-based practice of clinical skills, along with structured opportunities for self-assessment and peer and tutor feedback.

## INTRODUCTION

Clinical skills are an indispensable part of health professionals’ practice ensuring patient safety.^1^ Teaching these clinical skills to undergraduate medical students is a key component of the medical curriculum.^1^-^3^ Traditionally, it is taught through simulations and real patients using various approaches in the undergraduate medical curriculum. Simulation-based learning with mannequins, task trainers, or virtual reality environments allows students to practice skills in a controlled and safe setting. These simulations allow for teaching and assessment in a structured manner, while also providing opportunities to the students for deliberate practice.^4^ This training in controlled settings complements the workplace-based learning experiences with real patients under the supervision of experienced clinicians.^4^

Good medical practice^5^ and CanMEDS frameworks^6^ emphasizes being clinically skillful as one of the core competencies a physician must have. Individualized clinical skills teaching and feedback are a considerable problem due to resource constraints driven by the increasing number of healthcare students.^7^-^8^ Clinical teachers’ attitude, busy clinics, patient load, culture shift and workplace environment also pose problems.^9^ Absence of active participation of students in learning clinical skills, lack of direct observation and constructive feedback opportunities, failure to define clear objectives with a lack of reflection and discussion, make clinical learning more challenging. Ironically, many clinicians engage in clinical teaching without formal training and preparation.^10^ This needs attention, as the lack of necessary competencies by the graduates will impact healthcare and patient safety.

Student-centered approaches such as Team-based learning (TBL) and Problem-based learning (PBL) are extensively used for teaching the cognitive domain in the pre-clinical years of medical education. These approaches foster collaborative skills and critical thinking by engaging students to analyze and solve clinical problems. Little attention has been paid to the development of student-centered and peer-assisted learning approaches in the clinical phase of undergraduate medical education.^11^ A Team-Based Learning (TBL) approach has three distinct phases i.e., preparation before class, individual and group readiness assurance test (IRAT, TRAT) employing Multiple Choice Questions (MCQs), tutor clarification and application exercises.^12^ TBL increases students’ motivation, engagement and satisfaction. It promotes active learning and teamwork skills that are crucial for a good physician.^13^ In a few studies, its use in clinical years was investigated, however, it was limited to cognitive domain and acquisition of non-technical skills.^14^

TBL has not been used for teaching psychomotor domains and this is what we are going to explore in this study. We adapted TBL for teaching the psychomotor domain by providing resource materials such as clinical skills demonstration videos, readings and other online resources before the class. The MCQs, which are traditionally used for assessment of the cognitive domain, were replaced with Objective Structured Clinical Examination (OSCE) to assess the psychomotor domain^15^ during iRAT and tRAT. The iRAT was followed by self-assessment by the learner and the tRAT was followed by feedback from peers on clinical performance, with the tutor adopting an observational role and offering feedback as needed. The current study assessed the impact of this adapted Team-Based Learning approach with OSCEs for teaching clinical skills training to undergraduate medical students.

## METHODOLOGY

This quasi-experimental study with a crossover design was carried out from August 2021 till May 2022 at HITEC-Institute of Medical Sciences (IMS), Taxila, Pakistan (Ref no: ERC/36A/2021; Dated: 19 October 2021). It is registered at clinicaltrails.gov with registration#NCT06068062. A total of 80 medical students who fulfilled the inclusion criteria and provided informed consent to participate were enrolled.

### Inclusion criteria:

Willing students of the 4^th^ year MBBS HITEC-IMS Taxila who passed their 3rd professional exam were included in the study.

### Exclusion criteria:

The students who failed the professional exam and had to appear in the remedial exam were excluded. The participants were randomly assigned to control and experimental groups through a lottery method.

**Fig.1:**
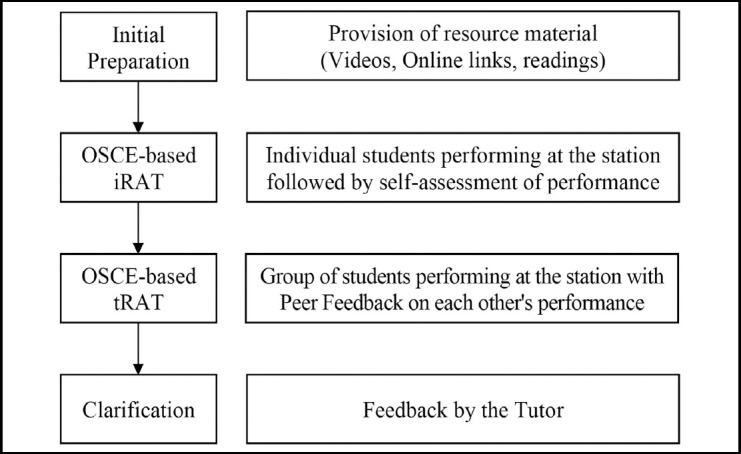
Adapted TBL using OSCE.

Two OSCE stations in obstetrics with pre-validated checklists were developed. Station one was Obstetric Examination and Station two was Eclampsia drill. The control group underwent standard teaching of clinical skills as routinely carried out in the department during the clinical clerkship of three weeks. In standard teaching, they are taught clinical skills in OPD and skills lab as per their timetable through a conventional teacher-centered strategy. The experimental group underwent an adapted Team-Based Learning (TBL) with OSCE-based iRAT and tRAT. The iRAT was followed by self-assessment by the learner and the tRAT was followed by feedback from peers on clinical performance, with the tutor adopting an observational role and offering feedback as needed (Fig.1).

Both groups had a pre-test at the start of the study in the form of two OSCE stations with pre-validated checklists to check their baseline knowledge. Post-test on the same stations was taken after standard teaching for the control group and an adapted TBL for the experimental group. In each group, the students were informed that it was a learning experience and that the scores would not affect their summative examination.

**Fig.2:**
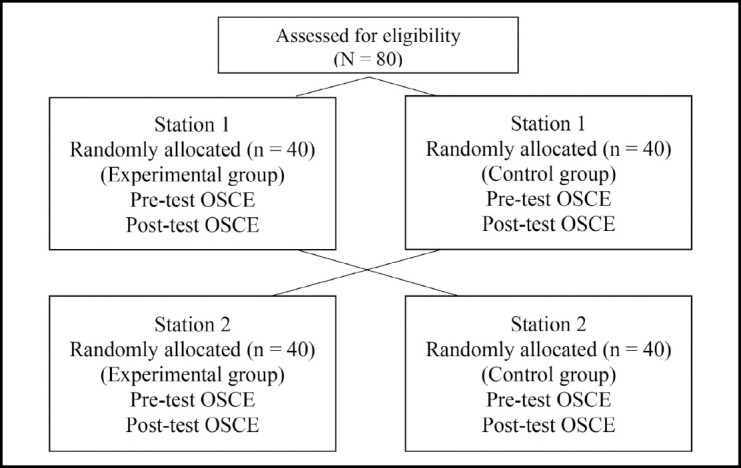
Study Design.

Both the experimental and control groups were provided with clinical skills demonstration videos, readings and other online resources before the class. Both the groups had an opportunity for feedback from the tutors. In the simulation center, each student in the experimental group performed the task individually (iRAT) with self-assessment and then the students performed the same task again in groups of five (tRAT), which was documented and assessed by their peers. Immediate feedback from peers was encouraged during tRAT. Students were also instructed to discuss among themselves and take turns from each other to make it a group activity with peer feedback in performing the task (tRAT). The performance was observed by a tutor, who adopted an observational role and offered feedback as needed (Fig.1). To control for potential skill and group effects, participants crossed over: the experimental group for obstetric examination served as the control group for eclampsia drill and vice versa. (Fig.2).

A pre-validated checklist (15 marks each) was used to evaluate the performance of students by trained examiners, before and after the intervention. To avoid any bias, it was ensured that the examiners who were part of this activity were not involved in the summative assessment. For both the control and experimental groups, pre- and post-test scores were documented. Descriptive statistics were reported as mean ± SD and inferential statistics were applied to evaluate the statistical significance of changes in scores. A paired sample t-test was applied to compare the mean scores of pre- and post-tests on each station for each group. An independent sample t-test was applied to compare pre-test and post-test mean scores of the control and experimental groups on both OSCE stations. Effect sizes were calculated using Cohen’s d.

## RESULTS

All participants were medical students of the 4^th^ year MBBS (n=80) at HITEC-IMS Taxila. There were no dropouts from the study. There were 42 females (53%) and 38 male (47%) students. Their age ranged from 23 to 25 years.

Pretest OSCE scores showed no significant difference between groups, confirming baseline equivalence. A statistically significant difference (p<0.001) was noted in the mean post-test scores of the experimental group (13.96 +0.62) and control group (10.34 +0.61) on OSCE station one. On OSCE station two, the difference between the mean post-test score of the experimental group (13.60 +0.52) and the control group (8.45 +0.42) was also statistically significant (p<0.001). The effect sizes were large and indicated a strong impact of the intervention on students’ performance (Table-I).

**Table-I:** Performance of control and experimental groups on both OSCE stations.

	Groups	Assessment	Mean	SD	Pre- and Post-test comparison (p-value)	Post-test comparison across groups (p-value)
Station 1	Control Group (n=40)	Pre-test	7.07	+0.60	<0.001	<0.001
		Post-test	10.34	+0.61		
	Experimental Group (n=40)	Pre-test	7.16	+0.52	<0.001	
		Post-test	13.96	+0.62		
Station 2	Control Group (n=40)	Pre-test	4.90	+0.48	<0.001	<0.001
		Post-test	8.45	+0.42		
	Experimental Group (n=40)	Pre-test	4.84	+0.44	<0.001	
		Post-test	13.60	+0.52		

## DISCUSSION

A quasi-experimental study with a crossover design was employed to compare a standard teaching strategy with an adapted Team-Based Learning (TBL) with OSCE-based iRAT and tRAT for teaching clinical skills to undergraduate medical students. Baseline scores of both groups were similar at the beginning of the study, eliminating the effect of the group. Moreover, the groups were crossed over and two different skills were taught to control for potential skill and group effects. The experimental group performed significantly better than the control group. Based on the findings, we believe that teaching clinical skills can be effectively achieved by incorporating the concepts of team-based learning using OSCE in a simulated setting. Patient fatigue is refuted by using simulations in skill labs, which is now highly recommended for patient safety and ethical reasons.

The adapted TBL approach using OSCEs is a student-centered strategy, based on principles of constructivism and adult learning, which encourages active construction of knowledge by the students without absolute reliance on the tutors for mere transmission.^16^-^17^ By embedding simulation and peer feedback, the model operationalizes Kolb’s experiential learning cycle, allowing students to move from concrete experience to reflective observation, conceptualization and active experimentation.^18^

Our findings resonate with Chen et al.’s meta-analysis, which demonstrated that TBL is more effective than traditional methods in improving learning outcomes.^19^ Similarly, Sattar et al. highlighted the benefits of flipped classrooms in fostering meaningful engagement among medical students.^20^ Objective Structured Clinical Examination (OSCE) is an effective way not only for summative assessment of medical students but also as a method of learning^21^ as also used in the current study. While innovations such as group OSCE,^22^ TOSCE and TOSBA have sought to enhance formative assessment, the OSCE-integrated TBL model offers a pragmatic balance between authenticity and feasibility. Singleton et al. validated the TOSCE using standardized patients as a formative assessment tool,^23^ while Rais et al. demonstrated the utility of TOSBA in bedside learning.^24^ These methods require an authentic clinical environment and are dependent on the skills of facilitators and are affected by patient fatigue and a limited pool of examiners/tutors, which can affect their quality.^25^ Compared to these, our model emphasizes self-assessment (assessment as learning), peer review and feedback within their groups (peer teaching) and may not need teaching a single skill to each student individually by the tutors/staff, who will be adopting an observational role and offering feedback if and when needed. These features make it particularly relevant for institutions with limited faculty resources.

The adapted TBL with OSCE provides a scalable and effective model for equipping medical students with essential clinical skills. Peer-assisted learning (PAL) offers a feasible, resource-efficient strategy that enhances learner engagement while simultaneously reducing faculty burden and is found to be comparable to traditional faculty-led teaching in improving knowledge acquisition among medical students.^26^ In our study, OSCE was embedded in TBL as a formative teaching tool, not as a high-stakes summative assessment. Therefore, the purpose was educational enhancement rather than psychometrically generalizable competency measurement. A study by Tufail and Ashar also reported significant improvement in post-test and ward test scores, emphasizing that OSCE used in a structured, feedback-oriented format enhances learning outcomes, even when not designed as a comprehensive summative assessment.^27^ Similarly, literature has described the importance of constructive feedback in bridging the gap between actual and desired performance.^28^

Several strengths emerged from this approach of using adapted TBL with OSCE as it seems to foster student-centered learning by encouraging active participation, teamwork, reflection and peer feedback. It offers rotating students in groups, which may reduce faculty burden while maintaining individualized feedback. This technique integrates learning theories of constructivism, experiential learning and peer-assisted learning to support deeper engagement and skill acquisition. Such methods may additionally support struggling undergraduate learners by promoting engagement and collaboration in a safe learning environment.^29^ Institutions may consider adopting this approach to strengthen formative assessment and skill development, particularly in undergraduate medical curricula. Even though the study was conducted on the undergraduate students, we believe that it may also be beneficial for the postgraduates, who have higher abilities to self-assess, self-regulate and improve themselves.^30^

### Limitations:

This study was conducted in a single institute and with one cohort of undergraduate medical students. Moreover, this study implemented the adapted TBL approach for only two skill stations and the results may vary depending on the type of skills. Therefore, the findings may not be generalizable and further evaluation across a wider range of skills and health professionals is recommended. Also, the students’ enhanced performance could be due to the novelty effect of the intervention. Unequal participation within TBL groups may hinder some students’ learning opportunities. We only reported a single outcome measure i.e., OSCE score, which assesses short-term performance. Future research should explore long-term outcomes, including retention of skills, impact on patient care and adaptability across diverse educational settings.

## CONCLUSION

Adapted team-based learning integrated with OSCE enhances clinical skill development in undergraduate medical students while reducing faculty burden, offering a practical and scalable solution. The approach encourages both individual and team-based practice of clinical skills, along with structured opportunities for self-assessment, peer and tutor feedback. Future studies should qualitatively explore students’ perceptions of other non-tangible benefits, such as engagement, confidence and team collaboration.

### Author’s Contributions:

The study was conducted by Dr. Ayesha Akram’s under the supervision of Dr. Ahsan Sethi. Dr Ayesha collected the data and performed data analysis. Both authors were involved in conceptualization, study design, writing and reviewing the final draft.
